# 2-Pentadecyl-2-oxazoline alleviates anxiety-like behaviour and modulates the microbiota-gut-brain axis in obese mice

**DOI:** 10.3389/fphar.2026.1878488

**Published:** 2026-06-17

**Authors:** Nicola Opallo, Adriano Lama, Stefania Melini, Lorena Coretti, Federica Comella, Filomena Del Piano, Nicole Pia Navatti, Alessia D’Andrea, Carmen De Caro, Francesca Lembo, Maria Carmela Ferrante, Rosaria Meli, Giuseppina Mattace Raso, Claudio Pirozzi

**Affiliations:** 1 Department of Pharmacy, School of Medicine, University of Naples Federico II, Naples, Italy; 2 Task Force on Microbiome Studies, Federico II University, Naples, Italy; 3 Department of Veterinary Medicine and Animal Production, University of Naples Federico II, Naples, Italy

**Keywords:** 2-Pentadecyl-2-oxazoline, barrier integrity, commensal bacteria, elevated plus maze test, high-fat diet, neuroinflammation

## Abstract

A bidirectional relationship between obesity and anxiety disorders has been increasingly associated with neuroinflammation and dysregulation of the gut–brain axis. Here, we investigated the pharmacological effects of the N-palmitoylethanolamine oxazoline derivative 2-pentadecyl-2-oxazoline (C15OXA) in a mouse model of high-fat diet (HFD)-induced obesity, with particular attention to its central and peripheral mechanisms of action. Male C57Bl/6J mice were fed an HFD for 12 weeks and subsequently treated with C15OXA (30 mg·kg^-1^, p. o.) for 7 weeks. Behavioural, molecular, and microbiota analyses were performed to evaluate the effects of the compound. C15OXA significantly reduced anxiety-like behaviour in obese mice without affecting body weight, fat mass, or glucose tolerance. At the central level, C15OXA attenuated hippocampal neuroinflammation, as shown by reduced expression of COX-2, TLR4, NLRP3 and IL-1β. In parallel, C15OXA restored tight junction gene expression associated with blood-brain barrier integrity, and modulated unfolded protein response signalling. In addition, C15OXA enhanced markers of neurogenesis and synaptic plasticity. At the peripheral level, C15OXA treatment reduced colonic inflammation and improved gut barrier integrity. These effects were associated with a targeted reshaping of gut microbiota composition. In particular, C15OXA promoted the enrichment of butyrate- and menaquinone-producing bacteria, as taxa linked to beneficial metabolic functions. Overall, these findings suggest that C15OXA exerts anxiolytic-like effects associated with coordinated central and peripheral pathways involving the modulation of neuroinflammatory pathways, barrier integrity, and gut-brain axis signalling. This study provides novel pharmacological insight into the therapeutic potential of C15OXA for the treatment of obesity-associated neuropsychiatric disorders.

## 1 Introduction

Obesity and overweight are only now clearly recognised as pathologies, whose impactful consequences encompass cardiovascular disease, type 2 diabetes, chronic kidney disease, and cancer (Carbone et al., 2025; Kong et al., 2025). However, more recently neuropsychiatric complications have been also included in the detrimental aftereffects of patients with obesity (Panda et al., 2023). In individuals predisposed to mood disorders, obesity may act as an aggravating factor. This condition may favour the onset of psychiatric conditions, such as depression or anxiety, following adverse stimuli. However, a bidirectional association has been suggested between obesity and mood disorders (Panda et al., 2023; Friedman et al., 2024), even though the underlying mechanisms are not yet fully identified. Experimental findings in animal models have attempted to study and dissect the intertwined interactions between the neurobiological and metabolic pathways at play. Specifically, high-fat diet (HFD)-induced obesity has been associated to the gradual development of mood disturbances in mice, like anxiety and depression (Lama et al., 2021; Lama et al., 2022). Converging lines of evidence have clearly identified numerous central derangements, closely related to chronic peripheral inflammatory conditions. Specifically, obesity has been recognized as a pathological disorder characterized by low-grade peripheral inflammation, referred to as metainflammation, which is orchestrated by metabolic cells in response to nutrient and energy overload, mainly involving adipose tissue, the gut and its microbiota (Cani et al., 2008; Gregor and Hotamisligil, 2011; Leyrolle et al., 2021).

Chronic HFD feeding has been reported to disrupt gut homeostasis, through microbiota dysbiosis and impaired colonic function. Alteration in the gut microbiota, including reduced bacterial load and loss of microbial diversity, compromise gut barrier integrity. This condition facilitates the leakage of LPS and other harmful microbial-derived factors into the bloodstream, ultimately allowing them to reach the CNS (Cani et al., 2008; Torres-Fuentes et al., 2017). Here, the activation of Toll-like receptors (TLRs) directly triggers NF-κB activation, thereby providing the priming signal required for inflammasome activation. This process drives the expression of proinflammatory cytokines, contributing to neuroinflammation [reviewed by Fiebich et al. (2018)]. Neuroinflammation is implicated in crucial pathological processes, such as dysregulation of neurogenesis, disruption of blood-bain barrier (BBB) integrity, and the accumulation of misfolded proteins (Bordet et al., 2023; Adamu et al., 2024; Garcia-Dominguez, 2025).

In past years, evidence have demonstrated the protective effects of 2- Pentadecyl-2-oxazoline (C15OXA), the oxazoline derivative of N-palmitoylethanolamine (PEA), a natural compound isolated from green and roasted coffee beans. C15OXA displayed neuroprotective activity in different animal models, recapitulating pain behaviour (Gugliandolo et al., 2018), ischemic brain injury (Fusco et al., 2019), neurodegenerative diseases (Cordaro et al., 2018; Infantino et al., 2022) or cognitive impairment (Guida et al., 2024). Although structurally related to PEA, C15OXA exhibits both overlapping and distinct effects, which underlie mechanisms of action that can be either shared or different (Impellizzeri et al., 2016; Petrosino et al., 2017).

Notably, Belardo et al. (2022) demonstrated that C15OXA could limit both behavioural and metabolic alterations, secondary to social isolation-induced stress, highlighting its potential in the treatment of multi-organ disorders with metabolic and neuropsychiatric components. The multi-target profile of C15OXA was further indicated by its modulating effect on gut microbiota composition and abundance, that have been associated to the pro-cognitive activity in mice with vitamin D deficiency (VDD)-induced memory deficits (Guida et al., 2024).

On these bases, we investigated the effect of C15OXA on obesity-induced anxiety-like phenotype in mice fed a HFD, identifying central and peripheral mechanisms along microbiota-gut-brain axis.

## 2 Materials and methods

### 2.1 Ethical statement

Animal care and experimental procedures were carried out in compliance with International and National Law and Policies and approved by the Local Animal Care Office (Centro Servizi Veterinari, Università degli Studi di Napoli, Federico II, ITA) and the Italian Ministry of Health under protocol no. 320/2021-PR (approved on 17 May 2021). Animal studies were performed in compliance with the Italian D.L. (No. 26 of 4 March 2014) of the Italian Ministry of Health and the EU Directive 2010/63/EU for animal experiments, ARRIVE guidelines 2.0, and the Basel declaration, including the 3Rs concept.

### 2.2 Animals and treatments

Male C57Bl/6J mice (Charles River, Wilmington, MA, United States) at 6 weeks of age were housed in stainless steel cages in a room kept at 22 °C ± 1 °C with a 12:12 h lights-dark cycle. Animals were randomly allocated to three experimental groups (at least 16 animals for each group) as follows: a control group (STD) receiving chow diet and vehicle (1.5% carboxymethyl cellulose [CMC], p. o., by oral gavage); an HFD group receiving vehicle; and an HFD group treated with C15OXA (HFD+C15OXA, 30 mg·kg^-1^ daily p.o). The selected dose was based on previous pharmacological studies and preliminary pilot experiments performed in our experimental conditions. Standard chow diet (4RF18, 17% energy from fat) and HFD (D12451, 45% energy from fat) were purchased by Mucedola srl (Milan, Italy) and Research Diets Inc. (NJ, United States), respectively. The detailed composition of both diets was reported in Supplementary Table S1. C15OXA was provided by Epitech Group spa (Saccolongo, Padua, Italy) and it was dissolved in CMC (1.5%) for oral gavage. The treatments started 12 weeks after HFD feeding and lasted 7 weeks until week 19. Before sacrifice, behavioural analyses were performed by investigators blinded to treatment allocation in assigned behaviour room during the light phase cycle of mice (at least n = 12). No animals were excluded based on experimental outcomes. At the end of the experiment, after fecal sample collection, all mice were euthanized with isoflurane by inhalation, followed by cervical dislocation. Hippocampus and colon were collected, snap-frozen and stored at −80 °C for subsequent analysis.

### 2.3 Body weight and composition

Body weight was assessed weekly throughout the experimental period. At the end of the 7^th^ week of treatment before killing, bioelectrical impedance analysis (BIA) was performed to evaluate fat body composition using a BIA 101 analyzer modified for the mouse (Akern, ITA). Fat mass content was derived from the difference between body weight and fat-free mass.

### 2.4 Oral glucose tolerance test

Oral glucose tolerance test (OGTT) was performed on different sub-groups of mice (5 animals/each group) at the beginning of the 7th week of C15OXA treatment. Mice were fasted overnight (16 h), and glycaemia was measured before glucose challenge and upon glucose solution (1 g/kg) administration (30, 60, 90, and 120 min). During this test, mice had free access to drinking water. Blood glucose levels were determined using the glucometer One Touch Ultrasmart (Lifescan, United States). The area under the curve (AUC) related to glucose level during the time was calculated from time 0 as an integrated and cumulative measure of glycemia up to 120 min for all animals.

### 2.5 Open field test

The open field test (OFT) is one of the most used to obtain general information about locomotor activity and emotionality of animals (Stanford, 2007). Indeed, the experimenter can evaluate anxiety-like behavior by evaluating mice’s propensity to avoid open areas perceived as dangerous, namely, thigmotaxis. Briefly, mice were placed in an open-field arena and allowed to explore for 30 min. After each trial, the arena floor was cleaned with 70% ethanol to delete odor cues for the next subject. The movements of mice were recorded by the infrared video camera and analyzed by Any-maze-tracking software (Stoelting Co., Wood Dale, IL, United States). The evaluated parameters from the open field test were: 1) the percentage of covered distance in the central area [(distance travelled in the centre/total travelled distance) x 100]; 2) the number of entries in the central area; 3) total travelled distance (in m).

### 2.6 Elevated plus maze test

The aversion of mice to open spaces and high distances is also evaluated by elevated plus maze test (EPMT). The maze (Ugo Basile apparatus, Gemonio, Italy) included four arms (5 cm in width and 35 cm in length): two opposing arms enclosed from the sides by black walls (20 cm in height), defined as closed arms; the other two arms remained unclosed (open arms). The access sector to all four arms is the center, also defined as the intersection area for all four arms. The test started placing mice individually in the center of the apparatus, facing an open arm, and allowed them to explore the maze for 5 min. The number of entries in open and closed arms and the time spent in these zones were detected and measured by the Any-maze tracking software. An arm entry is defined as the entry of all four paws into an arm. The percent time spent in the closed arm is considered as the main variable commonly associated to an ‘anxiety-like’ state. Total distance travelled (m) and average speed (m/s) was also monitored to evaluate locomotor activity of mice.

### 2.7 Quantification of gene expression using real-time PCR

Total RNA, isolated from the hippocampus or colon (from at least n = 5 animals each group), was extracted using TRIzol Reagent (Bio-Rad Laboratories, Hercules, CA, United States), following the instructions of RNA extraction kit (NucleoSpin®, MACHEREY-NAGEL GmbH and Co., Düren, Germany). cDNA was obtained using High-Capacity cDNA Reverse Transcription Kit (Applied Biosystems, Foster City, CA, United States) from 2 μg total RNA. PCRs were performed with a Bio-Rad CFX96 Connect Real-time PCR System instrument and software (Bio-Rad Laboratories). The PCR conditions were 15 min at 95 °C followed by 40 cycles of two-step PCR denaturation at 94 °C for 15 s, annealing extension at 55 °C for 30 s and extension at 72 °C for 30 s.

Each sample contained 500 ng cDNA in 2X QuantiTect SYBRGreen PCR Master Mix and primers pairs to amplify NLR family pyrin domain containing 3 (*Nlrp3*, mouse QT00122458), IL-1β (*Il1b,* mouse QT01048355), Claudin 5 (*Cldn5*, mouse QT00254905), Occludin (*Ocln*, mouse QT00111055), IL-10 (*Il10*, mouse QT00106169), tight junction protein 1 (*Tjp1*, mouse QT00493899 Doublecortin (*Dcx,* mouse QT02521155), Early growth response protein 1 (*Egr1*, mouse QT00265846), marker of proliferation Ki-67 (*Mki67*, mouse QT00247667) (Qiagen, Hilden, Germany), in a final volume of 50 μL.

The relative amount of each studied mRNA was normalized to β−Actin (*Actb*, mouse QT00095242) or GAPDH (*Gapdh,* mouse QT01658692) (Qiagen, Hilden, Germany) as housekeeping gene, and data were analysed according to the 2^−ΔΔCT^ method.

### 2.8 Western blotting

Hippocampus or colon (from at least n = 5 animals each group) were homogenized, and total protein lysates were subjected to SDS-PAGE. The blot was performed by transferring proteins from a slab gel to nitrocellulose membrane at 240 mA for 60 min at room temperature. The filter was then blocked with 1X PBS (pH 7,4) and 5% nonfat dried milk for 60 min at room temperature and probed with anti- COX-2 (1:1000 Santa Cruz, Cat. No #sc-19999), anti- TLR4 (1:1000 Cell Signaling, Cat. No #14358) anti- BiP (1: 1000 Cell Signaling, Cat. No #3177), anti- phospho-Eif2α (1:1000 Cell Signaling, Cat. No #9721), anti-Eif2α (1:1000 Cell Signaling, Cat. No #9722). Western blot for anti-β-Actin (1:5000; Cat No. #A5441; Sigma-Aldrich) or anti-GAPDH (1:5000, Cat. No. #2118 Cell Signaling) was performed to ensure equal sample loading. The detection of filter was performed by ChemiDoc Imaging System (Bio-Rad Laboratories, Hercules, CA, United States). The original Western blots are reported in Supplementary Material.

### 2.9 Gut microbiota-based studies

Bacterial genomic DNA was extracted from frozen fecal samples (n = 6 animals each group) and processed as previously described (Cimmino et al., 2025). The obtained paired-end reads (2 × 250 bp) were demultiplexed and processed using the Quantitative Insights Into Microbial Ecology (QIIME2, version 2022.2) (Bolyen et al., 2019) software to generate amplicon sequence variants (ASVs) that were taxonomically classified using the SILVA reference database (Quast et al., 2013). The interactive visualization of identified taxonomic data was depicted using the Krona program (Ondov et al., 2011). Alpha diversity metrics, namely, Chao1, Simpson’s diversity, Shannon entropy, and Pielou’s evenness were employed to assess the intra-group diversity of bacterial communities. Beta diversity, specifically unweighted UniFrac distances, was used to investigate the inter-group diversity. Statistical significance for alpha and beta diversities was respectively determined using the Kruskal-Wallis test and the Analysis of similarities (ANOSIM). Analysis of the differential relative abundances of bacterial genera was performed using the Microbiome Multivariable Associations with Linear Models (MaAsLin2) package (10.18129/B9. bioc.Maaslin2). Taxa were filtered to retain only those with a mean relative abundance >1% in all experimental groups and present in at least 10% of samples. Relative abundances were centred log-ratio (CLR) normalised to account for compositionality. The false discovery rate (FDR) was controlled using the Benjamini–Hochberg procedure, and results with q ≤ 0.25 were considered significant according to the default threshold of MaAsLin2 for exploratory multivariable microbiome studies. Sequences of key ASVs extracted from the microbiota dataset were blasted against NCBI database to identify the species with the highest identity. To quantify and assess the functional contributions of gut microbiota to brain- related processes, we performed a gut-brain module (GBM) analysis. Predicted metabolic functions of the microbial communities were inferred using the PICRUSt2 (Phylogenetic Investigation of Communities by Reconstruction of Unobserved States, version 2.4.1) analysis based on our normalised ASVs table, producing a stratified metagenome contribution file. An unstratified database (GBMs.v1.0) was used for pathway mapping, providing a curated collection of gut microbial pathways implicated in the synthesis or degradation of neuroactive compounds (Valles-Colomer et al., 2019). GBMs were computed per sample and per taxon, and abundances were summed across taxa to obtain GBM-level profiles. A GBM was considered present if its predicted pathway coverage reached at least 70%, to account for missing annotations and incomplete sequencing data. Only modules present in at least 10% of samples were retained for analysis. GBM abundances were CLR-transformed, and linear models were fitted separately for each module to test for differences between experimental groups. Group means were estimated using the emmeans package, version 2.0.0; FDR correction was applied across all GBMs using the BH method, and significance was assessed at q ≤ 0.2.

### 2.10 Data and statistical analysis

Data are presented as mean ± SEM. All experiments, except for microbiota study, were analyzed using analysis of variance (ANOVA) for multiple comparisons followed by Bonferroni’s *post hoc* test, using GraphPad Prism 10 (GraphPad Software, San Diego, CA, United States). Effect sizes for one-way ANOVA comparisons were estimated using eta squared (η^2^). Post hoc power analyses were performed using G*Power software (version 3.2) based on the observed sample sizes, alpha level (0.05), and effect sizes. Normality was tested using the Shapiro-Wilk test. Bonferroni’s *post hoc* test was run when F was significant. Differences among groups were considered significant at values of p < 0.05.

## 3 Results

### 3.1 C15OXA effects on metabolic alteration and anxiety-like behaviour induced by HFD

**FIGURE 1:**
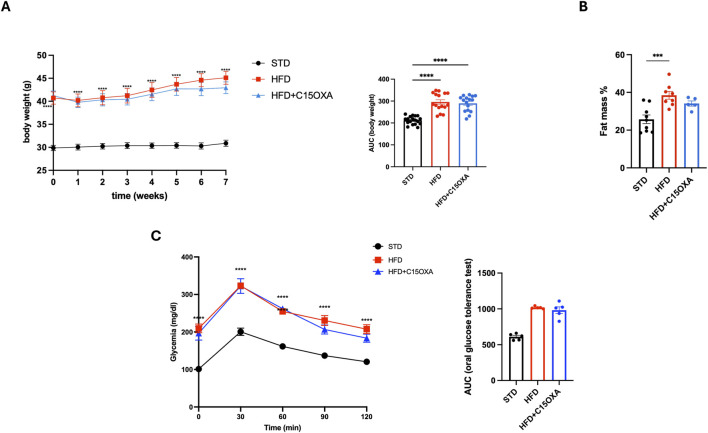
*In vivo* metabolic parameters. Body weight measured throughout the treatment period (0–7 weeks after 12-week HFD feeding) is reported along with AUC of weight during time (n = at least 16 each group) **(A)** Fat mass was measured by bioelectrical impedance analysis (n = at least 5 each group) **(B)** and OGTT is reported together with the AUC of glycaemia during time (n = 5 each group) **(C)** Data are presented as means ± SEM of animals from different groups.

HFD feeding led to a significant increase in body weight, that was weakly limited by C15OXA throughout the treatment period (Figure 1A). Consistently, cumulative body weight during the treatment time, evaluated as AUC was similar between untreated and C15OXA-treated HFD animals (Figure 1A). Accordingly other metabolic parameters, including fat mass, and glucose tolerance, deeply altered in HFD fed mice, were not modified by C15OXA treatment (Figures 1B,C).

**FIGURE 2:**
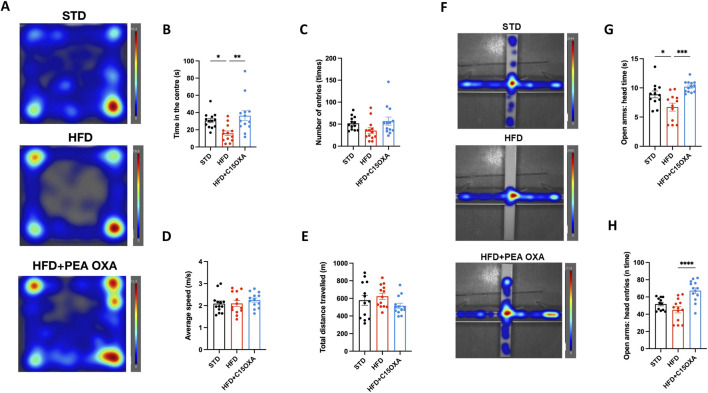
Effect of C15OXA on behavioral alterations induced by HFD. A representative heatmap made during the open field test and showing the anxiolytic effects of C15OXA compared to the thigmotaxis expressed by obese mice **(A)** (n = 11–14 each group). The time spent in the centre of the arena **(B)** the number of entries **(C)** the average speed **(D)** and the total distance travelled **(E)** were analysed. The anxyolitic properties of C15OXA were confirmed by Representative heatmap made during the Elevated Plus Maze test and showing the preference of untreated obese mice and HFD+OXA group for closed or open arms respectively **(F)** (n = 12–13 each group). Here, we evaluated the time spent **(G)** and the number of entries in open arms **(H)** Data are shown as mean ± SEM reaching the significance at P < 0.05.

In the OFT (Figure 2A), obese mice showed increased thigmotaxis and reduced latency to enter the central zone of the arena, as demonstrated by the significant reduction of time spent in the centre (Figure 2B). One-way ANOVA revealed a significant treatment effect on this parameter (F(2,36) = 7.151, p = 0.0024, η^2^ = 0.28), indicating a large effect size. This anxiety-like behavioural phenotype was attenuated by C15OXA treatment, which increased the exploration of the central area. No significant differences among groups were observed in the number of entries into the centre area, in average speed and total distance travelled (Figures 2C–E).

To confirm the protective effect of C15OXA on anxiety-like behavior of obese mice, we performed the EPMT, which determines the aversion of anxiety-like behavior in mice to open and elevated areas (Figure 2F). After HFD feeding, the obese mice showed a reduced time (Figure 2G) and a trend of reduction in the number of entries (Figure 2H) in open arms compared with STD mice. The anxiety-like behaviour induced by HFD feeding was significantly attenuated by C15OXA treatment, as evidenced by the increased time spent and number of entries in open arms (Figures 2G,H). One-way ANOVA revealed significant treatment effects for both time spent in open arms [F(2,34) = 11.26, p = 0.0002, η^2^ = 0.40] and number of open arm entries [F(2,35) = 13.62, p < 0.0001, η^2^ = 0.44], both indicating large effect sizes.

### 3.2 C15OXA lessens neuroinflammation and modulates BBB-related tight-junction gene expression in hippocampus of obese mice

**FIGURE 3:**
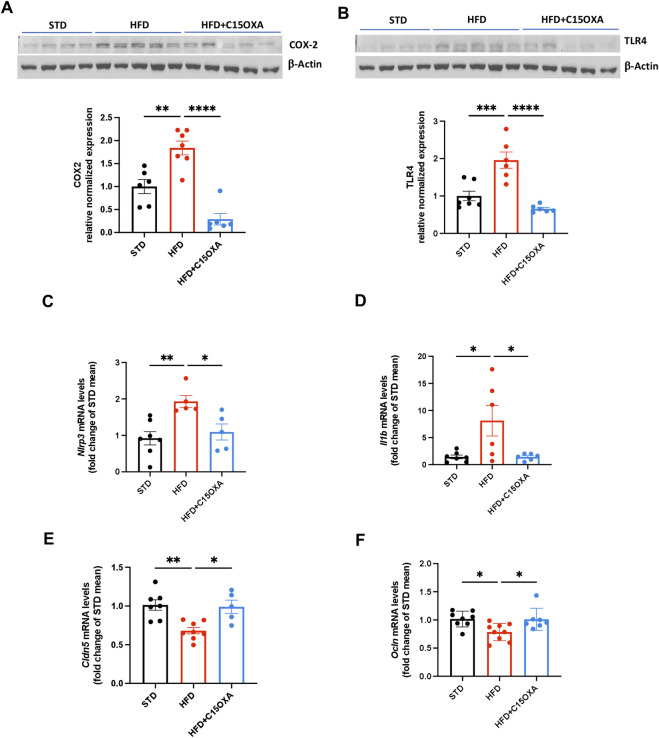
C15OXA reduces hippocampal inflammation and restored tight-junction expression in obese mice. Here we evaluated the protein expression of COX-2 **(A)** and TLR-4 **(B)** as inflammatory markers, by Western blot analyses (n = 6-7 each group). Moreover, the gene expression of the inflammasome *Nlrp3* **(C)** *Il1b* **(D)** and the tight junctions *Cldn5* **(E)** and *Ocln* **(F)** was assessed by Real-Time PCR (n = 5–9). Data are showed as mean ± SEM reaching the significance at P < 0.05.

The inflammatory patterns were evaluated in the hippocampus of mice from all experimental groups. HFD feeding enhanced the protein levels of COX-2 and TLR4 that were normalized by C15OXA treatment (Figures 3A,B). Moreover, C15OXA countered the increase of *Nlrp3* and *Il1b*, normalizing their mRNAs (Figures 3C,D). The transcriptional levels of the tight junction proteins claudin 5 and occludin, which were significantly reduced in untreated obese mice, were restored by C15OXA treatment, suggesting its potential beneficial effect on BBB integrity (Figures 3E,F).

### 3.3 C15OXA increases neurogenesis markers and counteracts ER stress in the hippocampus of obese mice

**FIGURE 4:**
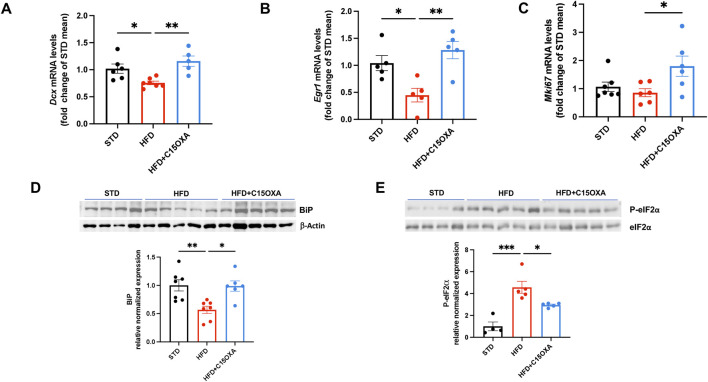
C15OXA restores neurogenesis and prevents the ER stress activation in hippocampus of obese mice. Gene expression of *Dcx* **(A)** *Egr1* **(B)** and *Mki67* **(C)** is shown (n = 5–7 each group). Protein expression of BiP **(D)** and the phosphorylation of EIF2α **(E)** were analysed by Western-Blot (n = 4–7 each group). Data are shown as mean ± SEM reaching the significance at P < 0.05.

To assess the effect of C15OXA on hippocampal neurogenesis, we evaluated the expression of *Dcx*, *Egr1,* and *Mki67* from mRNA extracts. C15OXA treatment normalized the HFD-induced reduction in *Dcx* and *Egr1* transcription (Figures 4A,B) and upregulated *Mki67* (Figure 4C). ER stress in hippocampus from all groups was also investigated. HFD induced a marked reduction in Bip/Grp78 protein expression, whereas C15OXA treatment restored the protein levels (Figure 4D). Consistently, C15OXA downregulated eIF2α phosphorylation (Figure 4E), indicating a marked attenuation of unfolded protein response pathway activation.

### 3.4 C15OXA counteracts colonic inflammation and permeability

**FIGURE 5:**
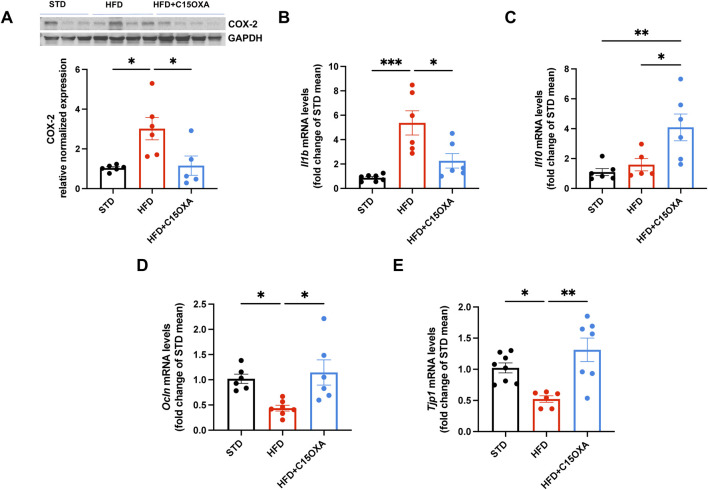
C15OXA attenuates gut inflammation and improves colonic integrity in HFD mice. Protein levels of COX-2 were evaluated by Western blot analysis **(A)** (n = 5–6 each group). C15OXA modulated mRNA levels of *Il1b*, *Il10*, *Ocln*, and *Tjp1* in colon of HFD group **(B–E)** (n = 5–7 each group). Data are presented as mean ± SEM reaching the significance at P < 0.05.

HFD feeding caused an increase in the levels of proinflammatory mediators, such as COX-2 enzyme expression and IL-1β (Figures 5A,B). The treatment with C15OXA dampened gut inflammation, significantly reducing both inflammatory factors and increasing the protective anti-inflammatory IL-10 transcription compared to HFD mice (Figures 5A–C). Moreover, C15OXA improved colonic integrity, as shown by restored transcription of the tight junctions, *Ocln* and *Tjp1* (Figures 5D,E).

### 3.5 C15OXA induces shifts in gut microbiota composition altered by HFD in obese mice

**FIGURE 6:**
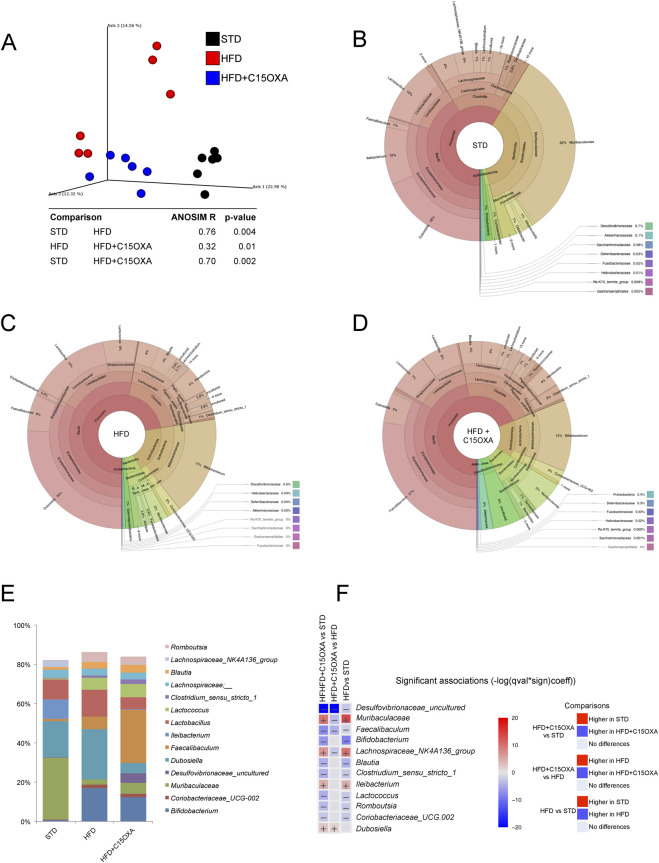
C15OXA reshapes gut microbiota composition of obese mice. Principal Coordinates Analysis (PCoA) based on unweighted UniFrac distances **(A)**. Each point represents the gut microbiota composition of one sample. The associated table displays the Analysis of similarities (ANOSIM) results testing differences among groups. Taxonomic composition of gut microbes in each group described using Krona **(B–D)**. From the inside to the outside, the circle represents the taxonomic levels of phylum, class, order, family, and genus. The size of the fan reflects the relative abundance of different taxonomic units. Data represent average values from sample groups. Stacked bar plot of relative abundance of bacterial genera with relative abundance >1% **(E)**. Differential abundance of gut bacterial genera with mean relative abundance >1% identified by MaAsLin2 as significant in HFD+C15OXA with respect to STD and HFD **(F)** (p-values were adjusted for multiple testing within each set of comparisons using the Benjamini–Hochberg procedure; Genera with q < 0.25 are considered significant).

To evaluate the impact of C15OXA on gut microbial communities, 16S rRNA gene sequencing targeting the V3–V4 hypervariable regions was performed on faecal DNA samples. Amplicon sequencing yielded a total of 1,337,497 high-quality reads across 18 samples, identifying 1,104 unique features. The sequencing depth ranged from 55,996 to 86,973 reads per sample, with a mean frequency of 74,305.4, ensuring sufficient coverage for robust taxonomic analyses. Unweighted UniFrac PCoA analysis showed a clear separation among the experimental groups, indicating a diet/treatment-related effect on gut microbioa composition (Figure 6A). Samples from the STD group clustered closely together, reflecting a consistent microbial profile. HFD samples were separated from STD and dispersed, indicating a distinct microbial community structure and a higher variability reflecting a reduced ecological stability (Figure 6A). Samples from the HFD+C15OXA group displayed decreased dispersion in β-diversity space, suggesting increased community stability, and formed a defined cluster located in an intermediate position between the other groups. These results were supported by ANOSIM, which revealed that C15OXA treatment induces a partial but statistically significant shift in gut microbiota composition distinct from HFD. Krona pie charts were used to visualize the taxonomic composition of the gut microbiota across the experimental groups at different taxonomic levels (Figures 6B–D). In the STD group, the microbial community was mainly dominated by Bacteroidota and Firmicutes while HFD group showed a marked remodelling toward an increase of Firmicutes and Actinobacteria with a concomitant reduction of Bacteroidota. In HFD+C15OXA group, while some HFD-associated alterations were still evident, a partial restoration of the relative abundance of major phyla was observed, with a trend toward recovery of Bacteroidota. Interestingly, within the Erysipelotrichaceae family belonging to Firmicutes, diet and treatment produced distinct genus-level shifts with *Ileibacterium* and *Dubosiella* enriched in STD, *Dubosiella* in HFD, and *Faecalibaculum* mainly increased in the HFD+C15OXA group (Figures 6B–D). *Faecalibaculum*, *Clostridium sensu stricto 1*, and Desulfovibrionaceae uncultured were the genera predominatly augmented by C15OXA treatment (Figures 6E,F). Moreover, C15OXA treatment partially restored the levels of common microbiota commensals, namely, Muribaculaceae and Lachnospiraceae_NK4A136_group, significantly increasing their abundance compared to HFD (Figures 6E,F).

In PICRUST2 prediction based on 16S rDNA sequence, the Gut–Brain Module (GBM) was used to map gut microbial-functions that have the potential to produce or degrade neuroactive compounds. GBM showed that bacterial metabolic pathways related to the metabolism of SCFAs (propionate degradation) and GABA synthesis significantly decrease (q < 0.05) in the HFD+C15OXA group and that C15OXA treatment induced an exclusive significant increase in the pathway related to Menaquinone synthesis (Vitamin K2) II (Supplementary Dataset 1).

## 4 Discussion

The present study identifies C15OXA as a multitarget modulator of obesity-associated neurobehavioral dysfunction, exerting coordinated actions across the microbiota-gut-brain axis. Beyond its anxiolytic-like effects, C15OXA mitigates key pathological processes, including neuroinflammation, BBB disruption, impaired hippocampal neurogenesis, and intestinal dysfunction, supporting a systems-level mechanism linking metabolic inflammation to behavioral alterations.

Obese rodents typically exhibit anxiety-like behaviour, largely attributed to HPA axis dysregulation and neuroinflammation (Lama et al., 2022). In our study, C15OXA treatment selectively attenuates anxiety-like responses without affecting locomotor activity, as shown in OFT and EPMT, thereby excluding nonspecific motor confounders. Rather than representing an isolated behavioural outcome, this phenotype likely reflects the normalization of neuroimmune and neuroplastic processes disrupted by chronic high-fat feeding.

While previous studies have highlighted the neuroprotective and anti-inflammatory properties of C15OXA in different central and peripheral pathological contexts (Impellizzeri et al., 2016; Cordaro et al., 2018; Fusco et al., 2019; Cordaro et al., 2020), its role in integrating metabolic, microbial, and neurobehavioral alterations remained poorly defined. Our findings extend this framework by demonstrating that C15OXA modulates behavioural outcomes in obesity independently of major metabolic improvements, suggesting a primary action on inflammatory and neurobiological pathways rather than systemic metabolic correction. Notably, in contrast to Belardo et al. (2022), who reported a resolution of metabolic alterations associated with social isolation of mice, our data reveal a clear dissociation between behavioural and metabolic outcomes in HFD-fed mice. This discrepancy may reflect the different experimental paradigms employed. Chronic HFD feeding is associated with a more severe metabolic burden characterized by persistent adiposity, metainflammation, and gut microbiota disruption. Therefore, under the present experimental conditions, the anxiolytic-like effects of C15OXA appear to be solely associated with the attenuation of inflammatory signalling across peripheral and central compartments rather than with weight loss or glycaemic improvement. Indeed, a marked anti-inflammatory effect was shown at the colonic level, suggestive of a systemic anti-inflammatory response possibly contributing to C15OXA central effect. Moreover, the attenuation of hippocampal TLR4-related inflammatory signalling is consistent with the modulation of endotoxemia-associated pathways widely implicated in HFD-induced metainflammation.

The hippocampus, beyond its established role in cognition, is also recognized as a critical hub for emotional regulation, exhibiting a pivotal role in the pathophysiology of cognitive and emotional impairment (Bannerman et al., 2003; McHugh et al., 2004). It has been widely demonstrated that HFD feeding triggers systemic metainflammation, which subsequently propagates to the central nervous system, driving neuroinflammatory responses (Hersey et al., 2021; Lama et al., 2022; Zou et al., 2023), including hippocampal proinflammatory marker upregulation that can impair the function of this specific brain region. In our experimental conditions, C15OXA suppressed hippocampal TLR4/NLRP3-driven inflammatory signalling in obese mice, as further supported by the decreased expression of COX-2 protein and IL-1β transcripts. In parallel, C15OXA restored tight junction gene expression, suggesting a functional coupling between neuroinflammation and barrier-related pathways. In the context of obesity-associated endotoxemia, this dual effect is particularly relevant, as it may limit the propagation of peripheral inflammatory cues into the central nervous system. These findings support a potential link between modulation of BBB-related markers and attenuation of neuroinflammatory processes. However, circulating markers of endotoxemia and systemic inflammation, including LPS, LBP, TNF-α, and IL-6, were not directly measured in the present study. Therefore, the contribution of systemic inflammatory mediators to the behavioural effects of C15OXA warrants further investigation.

Consistent with the interplay between inflammation and neuroplasticity in driving anxiety and depression (Anacker and Hen, 2017; Feng et al., 2025), we demonstrated that C15OXA counteracted HFD feeding-driven reduction in neurogenesis-associated markers, including *Egr1*, *Dcx*, and *Mki67* mRNA levels, indicating a recovery of hippocampal regenerative capacity.

Long-term obesity has been demonstrated to induce ER stress, mainly via the ATF4-CHOP axis, in the hippocampus, leading to a decrease in the number of processes in *Dcx*-expressing immature neurons specifically due to the loss of *Dcx* mRNA stability (Nakagawa et al., 2022). Some studies have highlighted the role of ER stress in worsening anxiety-like behavior in mice, pointing to a causative contribution of the adaptive unfolded protein response (UPR)-related pathways to abnormal apoptosis of brain cells (Liu et al., 2025). One of the UPR activation pathway begins with the dissociation of BiP/GRP78 from protein kinase RNA-activated (PKR)-like ER kinase (PERK), an ER stress sensor, leading to the phosphorylation of eIF2α, and ultimately contributing to neuronal apoptosis (Almanza et al., 2019). Here, C15OXA increased BiP expression while reducing eiF2α activation in the hippocampus of HFD animals. Given the established link between UPR activation and impaired neuronal function, this effect may contribute to restore cellular homeostasis and prevent maladaptive stress signalling in the brain during obesity.

In HFD mice, which typically display a profound perturbation of gut microbiota composition, the microbial changes observed upon C15OXA administration indicate a treatment-associated reshaping of the community, characterized not only by compositional changes but also by functional remodeling. Rather than globally reversing HFD-induced dysbiosis, the treatment promoted the enrichment of specific metabolically relevant taxa associated with host homeostasis.

Consistent with other studies addressing the modulatory effect of C15OXA on gut microbiota structure (Guida et al., 2024), we observed a marked shift in Erysipelotrichaceae membership from *Ileibacterium* and *Dubosiella* toward *Faecalibaculum*. Importantly, *Faecalibaculum* (*F. rodentium*, according to rRNA/ITs BLAST database) is a butyrate-producing commensal that supports gut epithelial integrity (Zhu et al., 2026), also enhanced in treatments ameliorating pathophysiological features of HFD mice (Fu et al., 2021; Guo et al., 2025). The concurrent increase of *Faecalibaculum*, *Clostridium sensu stricto 1*, and Desulfovibrionaceae uncultured (*F. rodentium, C. disporicum*, and *Mailhella massiliensis*, respectively, according to rRNA/ITs BLAST database) in C15OXA-treated HFD mice may reflect treatment-induced shifts in microbial cross-feeding interactions. Mucin-degrading Clostridia can enhance the SCFA production by *F. rodentium* and support the growth of sulphate-reducing Desulfovibrionaceae, while also potentially mitigating harmful levels of H_2_S produced by the latter (Marcelino et al., 2023).

Functional predictions inferred an enrichment of microbial pathways (i.e., among anaerobic Firmicutes and Desulfovibrionaceae) involved in menaquinone (vitamin K2) biosynthesis, a metabolite increasingly recognized for its neuroprotective and anti-inflammatory properties (Chatterjee et al., 2023). These predictive findings suggest a potential association between microbiota-derived metabolites and central nervous system function. Overall, these exploratory data suggest that C15OXA may reshape the gut microbiota of HFD-treated mice toward a profile potentially capable of influencing both intestinal and brain homeostasis. As functional predictions are inference-based, further integration with direct metabolite measurements and experimental approaches, such as faecal microbiota transplantation, will help to clarify whether the C150XA-induced gut microbiota profile contributes to the beneficial effects of the treatment on the pathophysiological phenotype of HFD mice.

A limitation of the present study lies in the lack of mechanistic intervention approaches to dissect causal pathways. Indeed, the overall 19-week HFD feeding, which also includes the 7-week chronic treatment of obese mice with C15OXA, does not allow for mechanistic investigations aimed at identifying the underlying specific molecular targets. Nevertheless, several available evidence supports a multimodal mechanism of action for C15OXA. The compound likely operates through converging mechanisms, including NAAA inhibition with subsequent modulation of PEA signalling (Petrosino et al., 2017), interference with TLR4-dependent inflammatory cascades (Facci et al., 2023), protean-like modulation of histamine H_3_ receptors (Boccella et al., 2021), and a mild regulatory effect on adrenergic signalling (Boccella et al., 2021; Infantino et al., 2022). Notably, the behavioural effects of C15OXA were not fully abolished in PPAR-α knockout mice (Petrosino et al., 2017), indicating only a partial dependence on this pathway. This evidence further supports a complex pharmacodynamic profile underlying the broad biological activity of C15OXA. Therefore, future mechanistic studies employing pharmacological inhibition strategies, transgenic models, and pathway-specific interventions will be necessary to dissect the relative contribution of the different signalling pathways involved in the effects of C15OXA. Importantly, only male mice were included in the present study in order to reduce hormonal and metabolic variability, mainly associated with HFD feeding. Considering the well-established sex-dependent differences in obesity-associated behavioural alterations, neuroinflammatory responses, microbiota composition, and endocannabinoid-related signalling, this may restrict the generalizability of the present findings. Therefore, potential sex-dependent effects of C15OXA should be specifically addressed in future investigations. Moreover, microbiota-related findings should be considered exploratory and hypothesis-generating, as functional analyses were inferred from 16S rRNA sequencing data using PICRUSt2 prediction.

**FIGURE 7:**
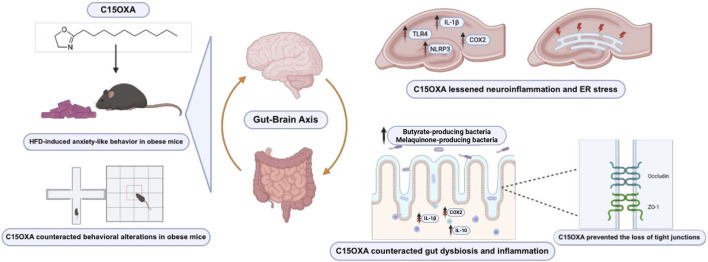
Schematic representation of the effects of C15OXA on the microbiota-gut-brain axis in HFD-induced obese mice. C15OXA modulates the microbiota-gut-brain axis in HFD-induced obesity by restoring intestinal and BBB-related tight junction gene expression, suppressing neuroinflammatory signalling, enhancing neurogenesis, and reshaping the gut microbiota.

In conclusion, C15OXA exerts anxiolytic-like effects in obesity associated with integrated modulation of neuroimmune signalling, barrier integrity, and microbiota-derived metabolic pathways (Figure 7). Considering the growing clinical relevance of obesity-associated neuropsychiatric disorders and the limited availability of targeted therapeutic strategies acting on multiple nodes of microbiota-gut-brain axis, C15OXA emerges as a promising pharmacological candidate worthy of further mechanistic and translational investigation.

## Data Availability

The datasets presented in this study can be found in online repositories. The names of the repository/repositories and accession number(s) can be found in the article/Supplementary Material.
