# Supplementary figures and images for: 2-Pentadecyl-2-oxazoline alleviates anxiety-like behaviour and modulates the microbiota-gut-brain axis in obese mice
Source: Front Pharmacol. 2026 Jun 17;17:1878488. doi: 10.3389/fphar.2026.1878488 (PMC13318990; doi:10.3389/fphar.2026.1878488)

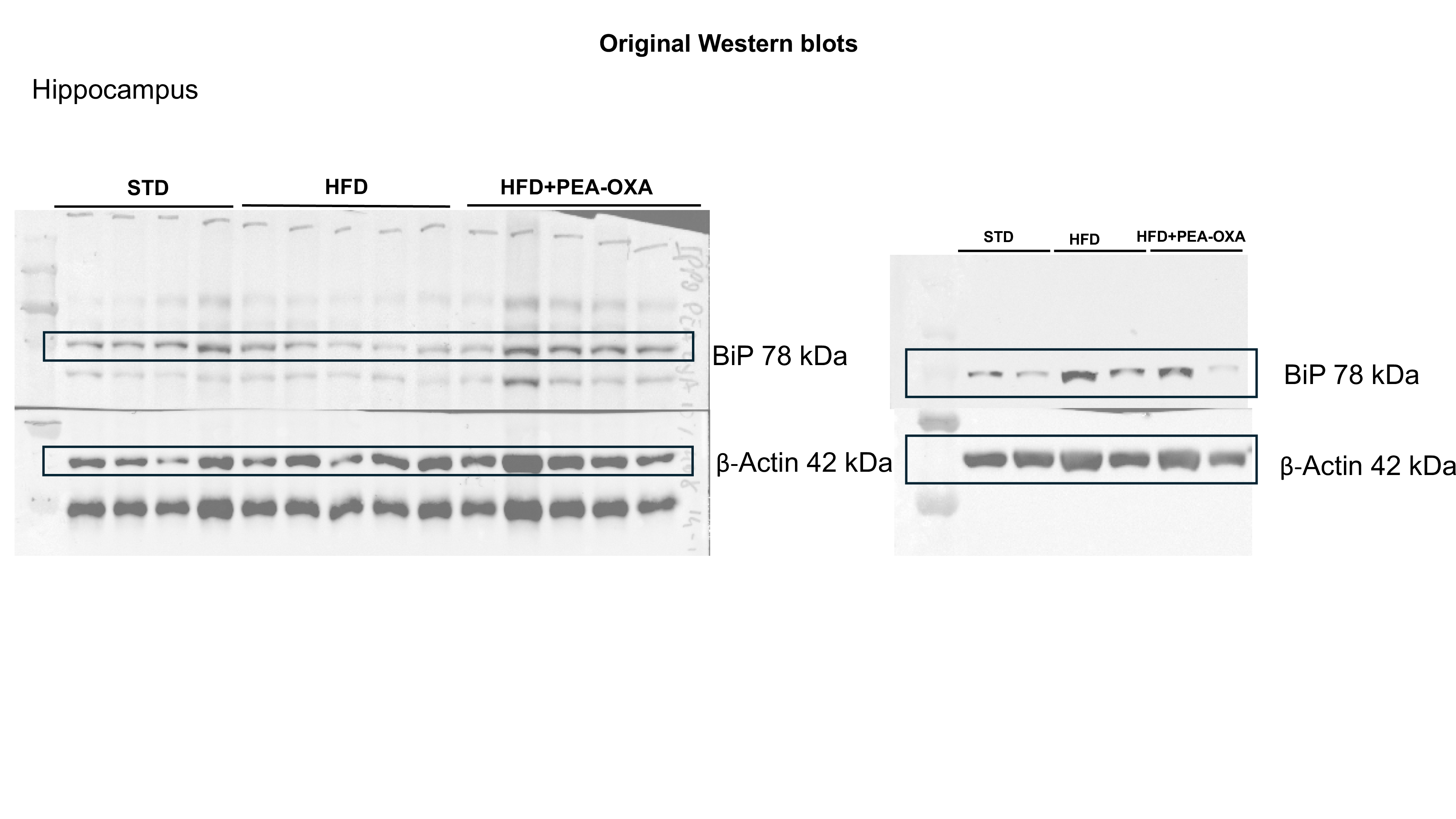

Supplement: Supplementary file 1 [file Image1.tiff]
