# Supplementary material for: 2-Pentadecyl-2-oxazoline alleviates anxiety-like behaviour and modulates the microbiota-gut-brain axis in obese mice
Source: Front Pharmacol. 2026 Jun 17;17:1878488. doi: 10.3389/fphar.2026.1878488 (PMC13318990; doi:10.3389/fphar.2026.1878488)
Supplement: Supplementary file 2 [file Table1.docx]

**Composition formulas and fatty acid profile of STD (Mucedola s.r.l., Milan, Italy) and HFD diet (Research Diets D12451 Inc., New Brunswick, NJ, USA).**

|  | **STD** | **HFD** |
| --- | --- | --- |
| **Diet composition** | **%** | **%** |
| **Protein** | 29 | 21,2 |
| **Carbohydrate** | 60,4 | 24 |
| **Fat** | 10,6 | 54,8 |
| **Energy, kJ/g** | 15,88 | 21,9 |
